# Integration of Discrete Simulation, Prediction, and Optimization Methods for a Production Line Digital Twin Design

**DOI:** 10.3390/ma16062339

**Published:** 2023-03-14

**Authors:** Damian Krenczyk, Iwona Paprocka

**Affiliations:** Department of Engineering Processes Automation and Integrated Manufacturing Systems, Silesian University of Technology, Konarskiego 18A Str., 44-100 Gliwice, Poland

**Keywords:** Digital Twin, Industry 4.0, integration, discrete event simulation, ant colony optimization, machine reliability, prediction

## Abstract

The integration of discrete simulations, artificial intelligence methods, and the theory of probability in order to obtain a high flexibility of the production system is crucial. In this paper, the concept of a smart factory operation is proposed along with the idea of data exchange architecture, simulation creation, performance optimization, and predictive analysis of the production process conditions. A Digital Twin for a hybrid flow shop from the automotive industry is presented as a case study. In the paper, the Ant Colony Optimization (ACO) algorithm is developed for multi-criteria scheduling problems in order to obtain a production plan without delays and maximum resource utilization. The ACO is compared to the immune algorithm and genetic algorithm. The best schedules are achieved with low computation time for the Digital Twin. By predicting the reliability parameters of the limited resources of the Digital Twin, stable deadlines for the implementation of production tasks are achieved. Mean Time To Failure and Mean Time of Repair are predicted for a real case study of an electric steering gear production line. The presented integration and data exchange between the elements of the smart factory: a Digital Twin, a computing module including an optimization, prediction, and simulation methods fills the gap between theory and practice for Industry 4.0. The paper presents measurable benefits of integration of discrete simulation tools, historical data analysis, and optimization methods.

## 1. Introduction

The development of manufacturing enterprises, especially automotive or ITC producers, is conditioned by the introduction of modern technologies and the requirement of continuous significant investments in research and development (R&D) [1]. In addition, there has been a recent trend in strengthening and synergy of two prominent concepts—Lean Management (LM) focused on quality and continuous improvement of processes and services, and Industry 4.0 focused on creating cyber–physical systems [2,3,4]. An increase in the level of production automation is observed with a simultaneous increase in the level of flexibility of production systems. Flexible systems adapt products to customer needs under mass customization conditions. This growth has resulted in an increasing amount of data that needs to be processed at each planning level, especially at the operational and tactical level. These factors influenced the formation of a new, revolutionary concept of industrial systems development, referred to as Industry 4.0. It includes production oriented towards cyber–physical systems integrating in a complete value chain (networks of interconnections), e.g., manufacturing, logistics, warehousing, and distribution. The production environment is enriched with new network systems (e.g., Internet of Things—IoT), wireless sensor networks (WSN), large data processing systems (Big Data), cloud computing solutions, embedded systems, and the mobile Internet [3,5]. The concept of Industry 4.0 and the implementation of the techniques and methods cited are presented, among others, in [4,5,6,7,8,9].

The successful implementation of the Industry 4.0 concept requires taking actions enabling the implementation of new methods of production and operation of enterprises characterized by three main features [10,11,12,13]:

- horizontal integration along the value chain network (e.g., free flow of information, finance, and materials from the customer through the manufacturer to the supplier, and vice versa);
- vertical integration (network production systems), related to the integration of hierarchical subsystems within the company (from the operational level with actuators or sensors, through the control level, to the production management level) in order to enable the creation of a highly flexible and reconfigurable production system;
- end-to-end engineering integration, related integration across the entire value chain to support product development and customization (from design to service).

These types of integrations along with the development of cyber–physical systems do not mean abandoning the Lean concept. On the contrary, the proposed development models [14] implement the principles of LM in all three forms of integration in Industry 4.0. Achieving a high level of flexibility and reconfigurability of production systems, as well as their autonomy, requires design and applications of smart machines and devices in combination with IoT techniques and cloud computing. Their computing, communication, and control capabilities, together with artificial intelligence methods and storage space, provide scalable computing solutions for the analysis of big data sets (smart factory), e.g., [12]. As a result, a cyber–physical system is created, in which the physical production system plays the role of access to data via sensors and communication systems operating in real time together with computing modules—the cyber layer (inside the resources and outside them—in the computing cloud). The analysis results and decisions in the feedback loops are sent back to the appropriate physical systems via the network [13,15].

This paper deals with issues related to the proposed approach to implementing vertical and horizontal system integration using computer simulation methods, production data exchange and transformation, and computer calculations for a Digital Twin of an automotive production system. Computer analyses are performed using artificial intelligence methods and the theory of probability as necessary elements of cloud computing in order to bridge the theory and practice.

### 1.1. A Digital Twin in the Implementation of Cyber–Physical Systems

The idea of using digital mapping of devices or entire production systems in the design, testing, and improvement process existed long before the concept of Industry 4.0 appeared. The concept of a twin in the sense of a prototype or copy of an existing object that reflected real operating conditions to simulate its behavior in real time emerged during NASA’s Apollo program. The “twin” of the spacecraft participating in the mission, remaining on the ground, was used to simulate its work. It used actual flight data to reflect its conditions as accurately as possible [16]. This concept was also used and developed in other large projects in the space and aviation industries. In the NASA Integrated Technology Plan, in the area of “Modelling, Simulation, Information Technology and Processing”—NASA Technology Map, 2010 and 2012—the term Digital Twin is defined as: “… an integrated multiphysics, multiscale, probabilistic simulation of an as-built vehicle or system that uses the best available physical models, sensor updates, fleet history, etc., to mirror the life of its corresponding flying twin” [6,8,15,16].

At the same time, in the area of product lifecycle management, Grieves [17] presented the concept of the Mirrored Spaces Model, consisting of three key elements characteristic of the Digital Twin: physical space, virtual space, and connection or interfaces between these spaces. This concept was developed by Grives, who incorporated the term Digital Twin [8,18]. The Digital Twin, in the context of the idea of a smart factory based on cyber–physical systems, corresponds to a virtual, dynamic model existing in the digital virtual space, which should be consistent with its physical counterpart in the real world. It should timely simulate its characteristics, behavior, functioning, and performance [8]. Thanks to the support of artificial intelligence and machine learning methods, as well as analytical software, based on feedback from the real system, a digital simulation is created on the information platform. Simulation models should automatically adapt to changes in the physical objects of the analyzed system. The requirement to automatically adapt the model to changes in the physical system applies primarily to situations where simulation models are used to support short-term operations management decisions. At the operational level, the time to find a solution is relatively short, which, in turn, results in a short time to update the simulation model [19,20,21]. Access to operational data and the use of communication components for data acquisition address several key issues of automatic adaptation of the simulation model. These include [19,21]: updating the system states in the simulation model using the latest data from the physical system, adjusting the simulation parameters, and updating the structure of the simulation model. The latter (the most challenging to perform) is related to the need to adjust the model structure at runtime in response to a structural change in the physical system [19,22]. These changes can result from a number of situations, from emergencies causing the need to modify routes and the use of alternative resources, through deficiencies related to interrupted supply chains at various levels, changes in demand, to the implementation of changes in response to the analysis of the previous iteration of optimization activities. The inability to automatically adapt simulation models to changes in the analyzed systems is, therefore, a significant obstacle to the possibility of using this type of method to support decision-making at the operational level. This, in turn, is a prerequisite for achieving the required high level of flexibility and reconfigurability of production systems, which is one of the goals of implementing the methods of the Industry 4.0 concept [12,15,16].

The application of the concept of creating Digital Twins based on simulation models in the area of production management is associated with implementation difficulties. These difficulties result from the time-consuming and cost-intensive process of building and updating models. These difficulties also increase with the increasing complexity of the systems depending on the number of workstations, the number of processes, the number of evaluation criteria, the number of reliability parameters, possible configurations of the production system, and the level of its flexibility. In this context, complex systems will consist of complex twins, which affect the complexity and cost of the Digital Twin [15]. In addition, the authors of [8] indicate the need to intensify the use of Digital Twin and big data technology in assembly systems for complex products. The authors of [9] distinguish four dimensions of modeling in the process of creating Digital Twins: geometry, physics, behavior, and rules. They indicate the need to implement a multidimensional connection of the simulation model with the production line in each dimension, as well as to analyze significance and mapping relationships between individual dimensions. They note that Digital Twin technology is an inevitable choice to shorten the development cycle and automation costs of production lines and realize the concept of smart manufacturing and services. Many articles point to the basic problem of building physical models of production systems equivalents in a virtual space, related to their high level of complexity. It has the effect of slowing down real progress in industry Digital Twin applications [8,9,12,13].

Another aspect related to the creation of smart factories is the need to ensure a high level of autonomy of cyber–physical systems. Implementation of this type of system will require access to very realistic models of real objects, reflecting their current state and logic of behavior (also in interaction with the environment). The basic method that can achieve this goal is the extensive use of simulation modelling, both in the design and planning phases and during other phases of the system’s life cycle [16]. From the point of view of computer simulation, the Digital Twin approach (digital objects with their structure, connections and existing meta-information and semantics) is the next phase in the development and application of modeling, simulation, prediction and optimization technologies [16]:

- since 1960—Individual application of simulation models: simulation limited to very specific problems and areas—carried out by experts.
- since 1985—Simulation Tools: a standard tool for answering specific design and engineering questions.
- since 2000—Simulation-based systems design: allows for a systemic approach to multi-level and multi-disciplinary systems with an extended range of applications.
- since 2015—The concept of a digital twin: simulation as the core functionality of the systems thanks to direct support throughout the entire life cycle—e.g., thanks to direct connection to operational data.

However, taking into account recent years, another step in the mentioned development process can be added:

- since 2020—Simulation-based cyber-physical systems: Digital twin solutions based on computer simulation supplemented with analytical modules based on artificial intelligence algorithms, probability theory and machine learning methods.

In literature a Digital Twin integration is classified in three levels [23,24]: digital model, digital shadow and Digital Twin:

- In a Digital Model, the data exchange between the physical and virtual model are fully manual, the changes updated in the physical system are not present in the digital system unless are manually updated and vice versa.
- In a Digital Shadow, the data flow from the physical world to a virtual system is automated, while the data flow from the digital system to the physical world is manual.
- In a Digital Twin, the bidirectional flow of data between the physical and digital system is automated. Each change to one system (physical or virtual) is updated in another.

In the related literature, there are many concepts of Digital Twin development, but the following stages are distinguished: reflection of the physical system into the digital systems, monitoring and controlling a Digital Twin, modeling and simulating the Digital Twin on the basis of the simulation results of data, federating the Digital Twin to optimize complex objects, acting autonomously, and recognizing and solving problems [25,26].

Currently, in most cases, simulation models of production systems are created “manually” by a person who knows a given modeling language or environment well, i.e., using expert knowledge. However, this process, especially for complex models, is slow and error prone. It is necessary to test the fit of the model to real data multiple times. The process of modifying an existing model, changing the operation execution logic, adding new resources, or reconfiguring the material flow is also very time-consuming. For this reason, the traditional modeling method is not effective enough in the process of creating Digital Twins supporting the implementation of the concept of cyber–physical systems. Integration with information systems and simultaneous data analysis are required, e.g., on the configuration of the production line, production resources, and process parameters.

The answers to these problems are developed methods of automatic or semi-automatic generation of simulation models. They are usually based on approaches using data and information obtained from ERP/MRP/MES/CAD/CAM systems used in enterprises. They can be divided into the following main categories [27]:

- Parametric approaches: models are generated based on existing simulation building blocks, stored in libraries that are selected and configured automatically or semi-automatically based on parameters.
- Structural approaches: model generation is based on data describing the structure of the system, usually in the form of factory layout data from relevant CAD systems.
- Hybrid knowledge approaches: combine both of the above approaches with artificial intelligence methods.
- The use of the generator usually requires the preparation of data downloaded from the company’s information systems. Therefore, their prior acquisition (usually in SCADA systems) and transfer to one of the source systems is required. These types of generators, creating virtual (digital) representations of real objects and systems, allow the speeding up the process of creating or updating a simulation model. However, they do not provide the supplementation of the simulation model with analytical modules that support the analysis of system behavior, which is the basic requirement for Digital Twins. Rocha et al. all [25] presented standards for data acquisition, digital representation of production hall elements, data control and visualization, and interoperability required for the development of a Digital Twin.
- A large amount of data obtained from production systems encourages the use of simulation and prediction methods. The trend to use artificial intelligence or machine learning is promoted by Industry 4.0 [28]. Taking advantage of the simulation, prediction, and optimization methods is crucial for achieving a high level of operation, flexibility, and reconfigurability in production systems.

### 1.2. Goals and Approaches

The aim of the article is to develop a method for generating simulation models so that they can be used in operational activities. A parallel goal is to achieve a high level of flexibility and reconfigurability of production systems, while ensuring the highest possible level of autonomy. 

The highlight of this paper is listed as follows:

The concept of a smart factory operation is proposed along with the idea of data exchange architecture, simulation creation, performance optimization, and predictive analysis of the production process conditions in order to obtain high flexibility of the production system.

Real production problems are more sophisticated in terms of optimization than academic flow shops and job shops. A Digital Twin is presented as a case study for the automotive industry. Mean Time To Failure and Mean Time of Repair are predicted for an electric steering gear production line.

Ant Colony Optimization (ACO) was developed for multi-criteria scheduling problems. A production plan with maximum use of resources and without delays is needed.

Taking advantage of the integration and data exchange between elements of a smart factory: a Digital Twin, a computing module (a cloud computing module), including optimization, prediction, and simulation methods, fills the gap between theory and practice for Industry 4.0.

In the next chapter, the mechanism of the smart factory operation is proposed along with a basic scheme for data exchange and the creation of simulation, optimization, and predictive modules. The third chapter presents an example of integration and data exchange of the operation of a smart automotive factory with elements: a Digital Twin, computing modules for optimization and prediction in the production process. The fourth section presents the discussion with the pros and cons of the proposed approach. The last section contains short conclusions and areas of further research work. 

## 2. Integration Method

**Figure 1:**
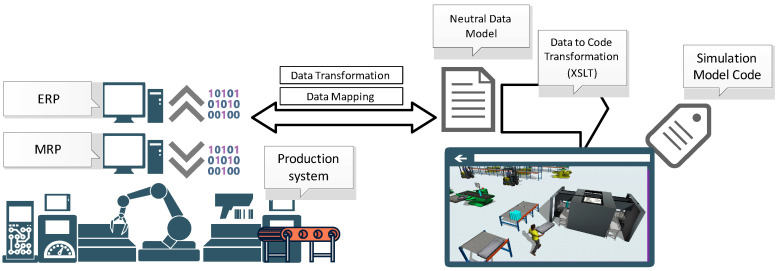
The simulation model generation process.

The approach proposed in this article is based on the development of the method of semi-automatic creation and automatic updating of simulation models using methods and data mapping and transformation with an intermediate step of data transformation in the form of a neutral data format. The idea of automating the construction and updating of simulation models consists of the transformation of data obtained from IT systems supporting business management at various levels and in various functional areas (ERP/MRP/MES/APS), through a neutral data model, to the programming code of the internal programming language of the simulation software. Diagram showing the idea of this method is shown in presented Figure 1 [29].

The simulation model generation process itself consists of several main stages. The first stage includes the acquisition of complete data required to prepare a neutral information model of the modeled production system. It uses data representation methods using the extensible XML markup language. Data exchange is then performed between the source data representation and the neutral intermediate data model. Thanks to this, the completed data required in the next stage are obtained, in which the data from the intermediate neutral model was directly transformed into the code in the internal programming languages of the simulation systems (the XSLT-based mapping and data transformation technique were used). The resulting code contains instructions in the internal programming language, creating resources that make up the manufacturing system, information resources in the form of tables, work schedules, or route data and parameters related to the scene, enabling the simulation process. The last stage is to load the finished model into the simulation system in order to verify it and conduct simulation experiments. More detailed information can be found in [29,30].

**Figure 2:**
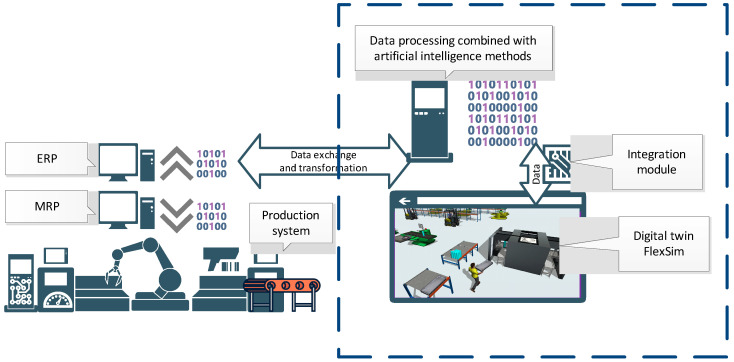
Simulation model integrated with analytical and optimization modules.

However, as mentioned earlier, in order for the simulation model to be useful as a Digital Twin, which is the central part of the cyber–physical system, the simulation model must be integrated with analytical and optimization modules. This part of the integration, which has been shown in Figure 2, together with the proposed algorithms for these modules, is addressed in this paper.

### 2.1. Integration Module 

Integration between the physical and virtual production process needs to be automated and all elements of the production stage need to be coordinated through artificial methods. The production process can be monitored using sensors and controllers and the real-time acquired operating data of the production process updated in the virtual twin, examining the impact of the change in the ACO beforehand. The Digital Twin is controlled by the best response achieved in the ACO. Integrated production and virtual processes can be driven by any change in the physical system (a new order arrival, a machine failure).

The production process is simulated using the ACO to estimate the efficiency depending on the sequence of running processes. When evaluating solutions, two objectives are taken into account: makespan and total tardiness. An ant represents a sequence of processes. In each iteration, the best ant is memorized. A list of achieved ants is introduced into the virtual simulation system to simulate the behavior of virtual model. Such a methodology helps to analyze and perform a comprehensive study of the performance and timeliness of the physical system in advance.

Apart from the system planning, the predicting of problems is an advantage in the presented methodology of integration for a Digital Twin. The optimized solution for the bi-criteria is achieved using the ACO, and the production plan is enhanced by including the maintenance aspect of the manufacturing process.

Virtual models of a production line are prepared in software “FlexSim” and ACO, while any problem detected in the physical production line is introduced in the systems.

Any information on machine failure or operation stops is collected by sensors and controllers and transferred to the SCADA system and Digital Twin. Next, historical information on the failure-free times of a machine is collected and analyzed for maintenance in the prediction module. 

**Figure 3:**
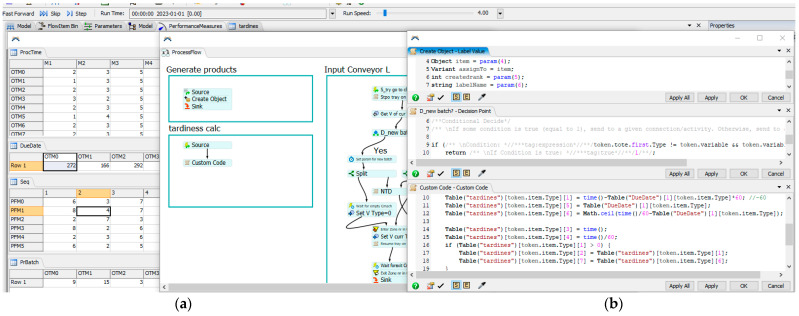
Integration modules directly in FlexSim. (**a**) data tables, (**b**) FlexScript modules.

Dedicated integrating modules/data exchange interfaces were created in order to be able to use the results generated by the ACO in the digital model prepared in FlexSim. These modules were written directly in FlexSim, using the internal FlexScript programming language. The exchange of data between the ACO and FlexSim takes place through messages in .txt files, from which data is then entered directly into tables in the FlexSim system (Figure 3a). Of course, in order for these data to be used during the simulation process (simulation experiments), it was necessary to develop integrating modules. Their task was to redirect the sent results so that they directly fed into the data of individual objects of the Digital Twin (workstations, generators of semi-finished products) (Figure 3b). Thanks to this, the best sequences obtained in the ACO were subjected to the verification process in the digital environment, in which the behavior of the real production system was mapped, also taking into account the subsystems of storage and inter-station transport together with operators involved in the processes of transport and manipulation.

### 2.2. Optimisation Module for Bi-Criteria Using ACO

The ant colony optimization (ACO) algorithm has become as a famous swarm-intelligence-based technique as the particle swarm optimization approach [31]. Swarm intelligence optimization algorithms are characterized by self-learning abilities, where each individual denotes a candidate solution in the entire search space. In swarm intelligence algorithms, the global search process is not random, but moves according to the position of the best individuals in the population. The sparrow search algorithm [32], gray wolf optimization algorithm [33,34], and Harris hawks optimizer [35] have been enjoying great interest. In this paper, the ACO algorithm was selected due to its popularity, easy implementation, self-learning ability, and simple framework.

The ACO structure consists of three parts: a master transition rule for the balance between exploration and exploitation, a global update rule for bi-objective optimization, a local update rule for makespan optimization. The Master Transition Rule determines whether the ant is focused on exploration (random path selection) or exploitation (attractive path selection) as it moves from point *r* to *s* (Equation (1)). If the ant is focused on exploration, it does not respond to information about solutions achieved in the past. This increases the likelihood of moving the ant to a more attractive area. If the ant is exploitative, the neighborhood of past, better solutions are followed. Ants prefer paths from the anthill to food rich in pheromone traces.

(1) $$S=\left\{ \begin{array}{l} \arg\underset{u\in N_{k}(r)}{\max}\{[\tau (r,u)]\cdot {\left[\eta (r,u)\right]}^{\beta }\;\},\;\;\mathrm{for}\;q\leq q_{0}\;\\ \;p_{k}(r,s),\;\;\mathrm{for}\;q>q_{0,} \end{array}\right.$$

where *q*_0_—a parameter, $q_{0}\in \langle 0,1\rangle$; *q*—a random number, q=〈0,1〉; τ—size of the pheromone trace on the edge *u*, between points *r* and *s*; $\eta =\frac{1}{\delta }$—reciprocal of the distance δ(r,u) representing a heuristic; β—a parameter of the relative importance between the pheromone trace and the reciprocal of the distance; $N_{k}\left(r\right)$—a set of points that ant *k* (located at point *r*) has not yet visited; and $p_{k}\left(r,s\right)$—a random variable selected according to:

(2) $$p_{k}(r,s)=\left\{ \begin{array}{l} \frac{[\tau (r,s)]\cdot {\left[\eta (r,s)\right]}^{\beta }}{\sum_{u\in N_{k}(r)}[\tau (r,s)]\cdot {\left[\eta (r,s)\right]}^{\beta }},\;\;\mathrm{for}\;s\in N_{k}(r)\\ \;0,\;\;\mathrm{for}\;s\notin N_{k}(r) \end{array}\right.$$

The ant will move to already discovered areas in the event of *q* ≤ *q*_0_. The most attractive task *s* for the ant is that with the shortest end time after *r*. In the event of exploration $q>q_{0}$, the ant discovers new areas. Point *s* is a random task from the following tasks after *r*. Each ant exploring a new area, if necessary, informs other ants about the attractiveness of the area, leaving behind a pheromone. The ant selects any available task, not just the shortest one.

The local update of the pheromone trail is performed in each iteration, for each ant, taking into account the makespan criterion. When building schedules, ants move between tasks, prioritizing tasks. The ants update the pheromone value even if they have not found the best schedule. The local update of the pheromone trail consists of decreasing the pheromone value for each pair of tasks in each iteration. Local updates of the pheromone trail prevent ants from gathering in only one sequence of tasks and introduces some variation in the resulting schedules:

(3) τ(r,s)←(1−ρ)⋅τ(r,s)+ρ⋅Δτ(r,s),

where *ρ*—a pheromone evaporation factor ρ∈〈0,1〉; τ(r,s)—an amount of pheromone on the sequence between tasks *r* and *s*; and Δτ(r,s)—reduction of the pheromone trace:

(4) $$\Delta \tau \left(r,s\right)=\tau_{0}=\frac{1}{n\cdot L_{nn}}$$

where *n*—a number of possible tasks to schedule after the task *r*; and *L_nn_*—minimum end time of two adjacent tasks.

The global pheromone update is performed for a relatively optimal task sequence. A relatively optimal sequence of tasks is the best one from the start of the algorithm or for each iteration:

(5) $$\tau (r,s)\leftarrow (1-\alpha )\cdot \tau (r,s)+\alpha \cdot \sum_{k=1}^{m}\Delta \tau_{K}(r,s),$$

where *α*—a pheromone evaporation rate, (1 − *α*) ϵ <0,1> a glow of the pheromone; τ(r,s)—the amount of pheromone for task pair *r* and *s*; *m*—a number of ants that have passed from point *r* to point *s*; and $\Delta \tau_{K}(r,s)$—an increase in the pheromone trace is calculated from:

(6) $$\Delta \tau_{K}(r,s)=\left\{ \begin{array}{l} \frac{1}{L_{K}},\;\;\mathrm{for}\;(r,s)\in L_{K}\\ \;0,\;\;\mathrm{for}\;(r,s)\notin L_{K} \end{array}\right.$$

where $(r,s)\in L_{K}$—edge belonging to the global best schedule; *K*—index of the ant that discovered the best sequence of tasks; and $L_{K}$—a weighted function of makespan and total tardiness of the schedule achieved for the best global sequence.

(7) $$L_{K}=0.5\cdot \frac{C_{max}^{}}{C_{max}^{*}}+0.5\cdot \frac{TT}{TT^{*}}.$$

where $C_{max}$—makespan of the global best schedule; $C_{max}^{*}$—makespan of the global worst schedule; TT—total tardiness of the global best schedule; and $TT_{}^{*}$ total tardiness of the global worst schedule. 

**Figure 4:**
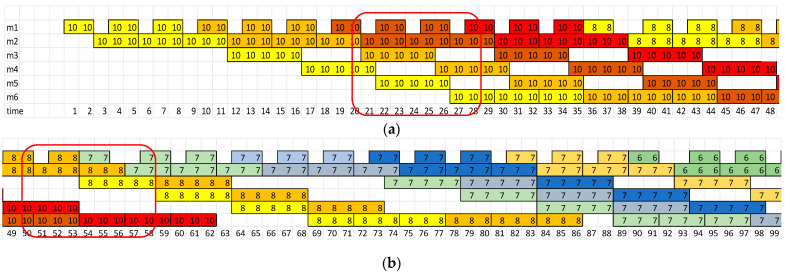
Gantt chart of the hybrid flow shop with a production batch and a transport batch (where m_1_–m_6_—machine number; 1–99 time unit [mins]; 10,8,7,6—order number). (**a**) parallel washing of elements in chambers m_3_, m_4_ and m_5_, (**b**) processing on machine m_6_ after a fully completed washing cycle.

The presented method supports the scheduling of tasks on limited system resources, so that the resources are maximally occupied, and the tasks are delayed as little as possible in relation to the deadlines required by customers. Scheduling algorithms are specific to real production systems with process-specific constraints. Consider a schedule for a hybrid flow shop where the production batch does not have to be equal to the transport batch. Restrictions apply:

- The flow of tasks through the first and second machines is typical for a flow system, a one-piece flow applies;
- The third, fourth, and fifth machines represent the chambers of the washing machine;
- The machine is equipped with an accumulative conveyor that can hold three blanks;
- The accumulation conveyor enables parallel operation in the chambers, independent of the end of the washing cycle of elements in other chambers (Figure 4a);
- When switching to a new job, the component wash cycle must be fully completed to allow the machine to be retooled (Figure 4b);
- The washing machine releases three items in each cycle, which are passed on to the next machine, where the flow of one item resumes.

A production plan with maximum use of resources without delays is needed. Maximum use of resources was assessed using the Makespan criterion (Cmax), and delays were calculated using the Total tardiness criterion (TT) (7). In addition, predictive schedules include the maintenance time of the critical machine in order to increase schedule stability.

The tuning of ACO parameters can improve the quality of implemented schedules. By lowering the *q*_0_ parameter, the ants pay too much attention to exploring new areas. A high value of *ρ* means that pheromone evaporates quickly, and already-found schedules with better values of Cmax and TT criteria are quickly forgotten by other ants. The number of ants and the number of iterations may not be enough to explore new areas or take advantage of the better schedule achieved in the past. Parameter *β* determines the relative importance between the pheromone trail and the reciprocal of execution time of two adjacent tasks. All parameters should be specified for a given scheduling problem. Parameters once specified may not be effective for other scheduling issues, including both size and type.

### 2.3. Problem Prediction Module 

Given the frequency of machine failures, it is important to identify all the factors that cause failures in order to avoid them in advance and prevent failure conditions. Each failure and failure circumstances are recorded in the SCADA system. Planning technical inspections of machines leads to a reduction in the number of costly repairs. The collected information is the basis for setting the dates of machine inspections and determining the scope of maintenance work. Scheduling machine maintenance leads to increased machine uptime and machine life. Increased uptime reduces the nervousness of the adopted schedule, and thus improves the stability of the entire production system. Determining the Mean Time To Failure (MTTF) also allows for early scheduling of the supply of machine consumables, and thus prevents disruption related to the lack of spare components in the warehouse.

Machine uptime depends on the phase of its life cycle. The life cycle curve of the machine is modeled by the traditional bathtub curve, although the damage intensity function can also take other forms [36]. The analyzed production process is a newly established factory. The machines are in the first phase of their life cycle. In this phase, the weakest elements are damaged at an early stage, and the errors that appear result from the fact that the employees learn how to operate the process and the system elements must adapt to the working conditions.

The failure-free operation times $X_{i,1},\ldots ,X_{i,N_{i}}$ of a machine are described by a Weibull distribution with the PDF (probability density function) of the form

(8) $$f_{i}\left(t\right)=\{\begin{array}{c} \lambda_{i}p_{i}t^{p_{i}-1}\exp\left(-\lambda_{i}t^{p_{i}}\right){,}_{}t>0,\\ 0{,}_{}t\leq 0, \end{array}$$

where $p_{i}>0,\;\lambda_{i}>0,$ thus parameters of the distribution depend on the number of period and are the same in each period separately. Here, *N_i_* denotes a random number of failures detected in (i−1)T,iT)[ ). As it is well known:

(9) $$EX_{i,k}={\left(\frac{1}{\lambda_{i}}\right)}^{1/p_{i}}\Gamma \left(\frac{1}{p_{i}}+1\right)$$

(10) $$EX_{i,k}^{2}={\left(\frac{1}{\lambda_{i}}\right)}^{2/p_{i}}\Gamma \left(\frac{2}{p_{i}}+1\right)$$

where $EX_{i,k}$—mean value of $X_{i,k}$; $EX_{i,k}^{2}$—second moment of $X_{i,k}$; and *k*—number of failures detected in month *i*, $k=1,\ldots ,N_{i}$.

At the end of each operation time, after a failure, a repair time begins immediately and so on. Repair times $Y_{i,1},\ldots ,Y_{i,N_{i}}$ collected for each month are supposed to be exponentially distributed with PDFs of the form:

(11) $$g_{i}\left(t\right)=\left\{ \begin{array}{l} \alpha_{i}\exp\left(-\alpha_{i}t\right){,}_{}t>0,\\ 0{,}_{}t\leq 0, \end{array}\right.$$

The analyzed production process is at the stage of improvement, the data is collected on the basis of six-month observations. Initial observations are made for one shift, then the second and third shifts are started in turn.

The condition for $p_{1}$ of Weibull distribution *R*(*t*) for the first past month is:

(12) $$n\sum_{k=1}^{n_{1}}x_{1,k}^{p_{1}}\ln x_{1,k}^{}-\left(\frac{n_{1}}{p_{1}}+\sum_{k=1}^{n_{1}}\ln x_{1,k}^{}\right)\sum_{k=1}^{n_{1}}x_{1,k}^{p_{1}}=0.$$

Having $\hat{p_{1}}$, the estimator for $\lambda_{1}$ is find using the Maximum Likelihood:

(13) $$\hat{\lambda_{1}}=\frac{n_{1}}{\sum_{k=1}^{n_{1}}x_{1k}^{\hat{p_{1}}}}$$

Having parameters $\hat{p_{1}},\ldots ,\hat{p_{i}}$ and $\hat{\lambda_{1}},\ldots ,\hat{\lambda_{i}}$ for each historical month, classical regression is used in order to predict parameters for the future period *i* + 1. The parameters of distribution *R*(*t*) for the next period are introduced into the equation of mean time between failures:

(14) MTBF=E{Xi+1,1+Yi+1,1}=(1λ^i+1)1p^i+1Γ(1p^i+1+1)+(1αi+1).

where Γ—Gamma distribution; and $\alpha_{i+1}$—parameter of the exponential distribution describing repair times.

## 3. Digital Twin Case Study

**Figure 5:**
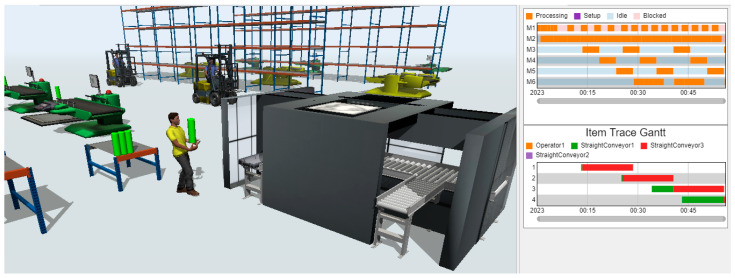
Digital twin model in FlexSim.

The Digital Twin project was developed for the area of the electric steering gear production line, namely: drilling and threading operations, as well as washing the steering gear housing. The washing operation is semi-automatic: loading and unloading of elements to be washed is performed manually, while the washing process and fluid regeneration through filtration is automatic with the need for periodic replacement of filter cartridges (Figure 5). The details are transported on specially designed pallets, each equipped with a mount for three independent details. The washing solution and rinsing water are electrically heated, and the tanks have level and temperature controls. The machine is equipped with visual signaling of contamination of filter cartridges. The machine is also equipped with four zones: pre-washing, main washing, and drying and blowing with compressed air. The parameters of the initial and main washing process (e.g., times of washing, blowing with compressed air) are set for each batch of a new type of casing. It is necessary to empty the details from the unloading area. The machine does not start the cycle if there are details on pallets in the unloading area. During the cycle, the operator loads the parts to be washed and unloads the washed parts. After the cycle is completed, the controller makes sure that all activities have been performed and goes into standby mode until the start button is pressed.

Each production process was described by the Operation Times Matrix OTM and the Process Flow Matrix PFM (Figure 3). The batch size of each production order is described in the Batch Size Vector = [9,15,3,12,9,6,12,6,9,12,15,6]. Production orders should be completed before the deadlines specified by the Due Date Vector = [272,166,292,178,302,190,200,200,300,250,220,290].

### 3.1. Problem Statement 

A production plan without delays and maximum resource utilization is needed. The problem is complex, as an increase in orders for finished products by customers has necessitated the activation of the second and third shifts. At the same time, on-time production is required. On-time production is possible if the need for intervention by the maintenance team can be foreseen and machine failure is prevented. Historical data from six months of operation is used to predict the time of failure of the washing machine (Table 1).

The simulation model is powered by sequences of production tasks, achieved by the ACO algorithm, and the predicted uptime of the washing machine, using probability theory. Integration and data exchange make the Digital Twin model a full exploration of the production line.

### 3.2. ACO Tuning

The ACO parameters to achieve the effects of on-time production and maximum utilization of the automotive company’s resources are tested. The goal is to obtain a set of parameters that yields the best schedules in the analyzed case study.

**Figure 6:**
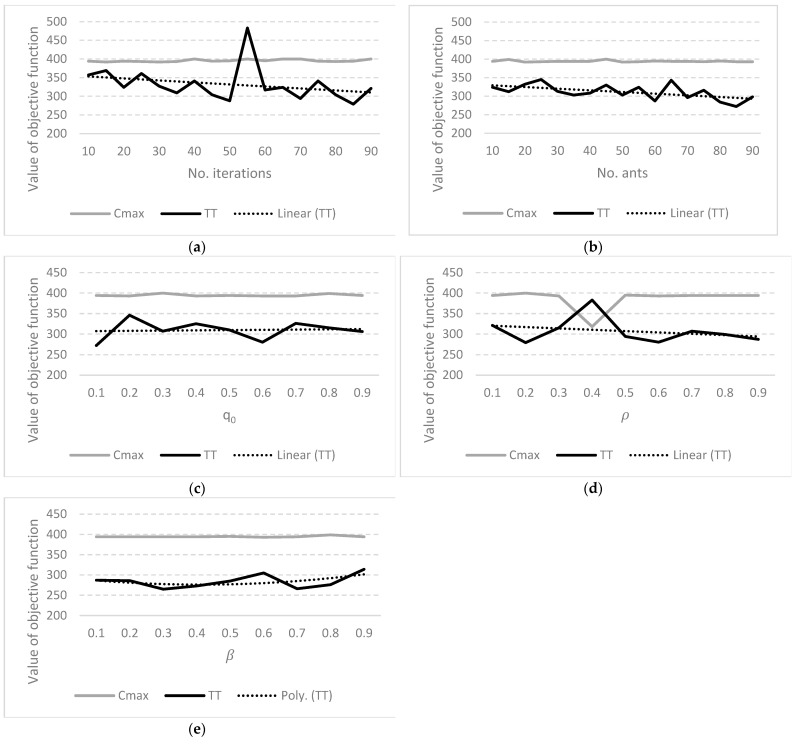
Results obtained by the ACO for changing parameters: (**a**) number of iterations, *E*; (**b**) number of ants; (**c**) *q*_0_; (**d**) *α* = *ρ* and (**e**) *β*.

Computer simulations were run for the parameters: relative importance between the pheromone trace and the reciprocal of the distance β={0.1,0.2,0.3,0.4,0.5,0.6,0.7,0.8,0.9}; pheromone pairing factor α=ρ={0.1,0.2,0.3,0.4,0.5,0.6,0.7,0.8,0.9}; number of ants, *K* = {10,15,20,25,30,35,40,45,50,55,60,65,70,75,80,85,90}; number of iterations, *E* = {10,15,20,25,30,35,40,45,50,55,60,65,70,75,80,85,90}; and parameter *q*_0_, which decides about exploration or exploitation selection by each ant, $q_{0}=\left\{0.1,0.2,0.3,0.4,0.5,0.6,0.7,0.8,0.9\right\}.$ The results obtained by the ACO are presented for the input parameters:

- {*ρ* = 0.6, β=0.1, *K* = 20, *q*_0_ = 0.5} and changing number of iterations, *E* (Figure 6a);
- {*ρ* = 0.6, β=0.1, *E* = 40, *q*_0_ = 0.5} and changing number of ants, *K* (Figure 6b);
- {*ρ* = 0.6, β=0.1, *K* = 40, *E* = 40} and changing parameter, *q*_0_ (Figure 6c);
- {β=0.1, *K* = 40, *E* = 40, *q*_0_ = 0.5} and changing pheromone pairing factor, ρ (Figure 6d);
- {*ρ* = 0.9, *K* = 40, *E* = 40, *q*_0_ = 0.5} and changing parameter, β (Figure 6e).

First, the influence of the number of iterations over the Cmax and TT of schedules was examined (Figure 6a). With the increase in the number of iterations, the chance of finding a better solution increased, taking into account the criterion of TT. The opposite phenomenon was observed for the Cmax. The objective functions were contradictory, i.e., the improvement of the evaluation criterion resulted in the simultaneous deterioration of the second objective function. 

The effect of the number of ants on the Cmax and TT of schedules was then examined (Figure 6b). With the increase in the number of ants, the chance of finding a better solution taking into account the TT increased. The probability was greater than in the case of the influence of the number of iterations. The effect of the number of ants on the Cmax was opposite to the effect on the TT.

Further simulations were carried out for the number of iterations = 40, and the number of ants = 40, as higher parameter values increased the chance of finding a better solution. The effect of the parameter *q*_0_ on Cmax and TT of schedules was not as important as the effect of the pheromone pairing factor *α* = *ρ* (Figure 6c,d). With the increase in the pheromone pairing factor, the chance of finding a better solution taking into account the TT increased (Figure 6d). The effect of both parameters on the Cmax was not crucial.

Since, the effect of parameter *q*_0_ was not as important as the effect of the pheromone pairing factor *ρ*, further simulations were carried out for *q*_0_ = 0.1 and *ρ* = 0.9. The choice of the values of the parameters *q*_0_ and *ρ* was conditioned by the achievement of the best schedules. The TT was 272, Cmax = 394 for the schedule described by the task sequence {7 9 1 3 5 6 0 4 2 8 11 10} obtained with *q*_0_ = 0.1. Exploring the space of solutions was important for the analyzed Digital Twin. The TT was 287, Cmax = 394 for the best schedule described by the task sequence {2 3 1 5 6 0 9 7 11 8 4 10} obtained for *ρ* = 0.9.

Finally, the effect of parameter *β* on the Cmax and TT of schedules was examined (Figure 6e). With the increase in the *β* parameter, the chance of finding a better solution taking into account the TT increased. The effect of the *β* on the Cmax is negligible. Not all scheduling problems could be assessed using only the Cmax criterion. In the analyzed case study, it was necessary to take into account the second criterion. The best schedule described by the task sequence {7 3 6 1 2 5 9 4 11 8 0 10} was obtained for *β* = 0.3 with the TT of 265 and Cmax = 394. The following set of parameters allowed to find the best schedules for the problem: {*β* = 0.3, *ρ* = 0.9, *K* = 40, *E* = 40, *q*_0_ = 0.1}.

The presented ACO algorithm was compared with a genetic algorithm (GA) [37] and an immune algorithm (IA) [38]. Computer simulations were run for changing iteration number, *E* = {10,30,50,70,90,160}, and changing population size, *K* = {10,30,50,70,90,160}: number of ants in the case of ACO, number of chromosomes in the case of GA and IA. The remaining set of parameters was {*β* = 0.3, *ρ* = 0.9, *q*_0_ = 0.1} for the ACO. Remaining parameters of the GA and IA was specified in [37,38], respectively. 

**Figure 7:**
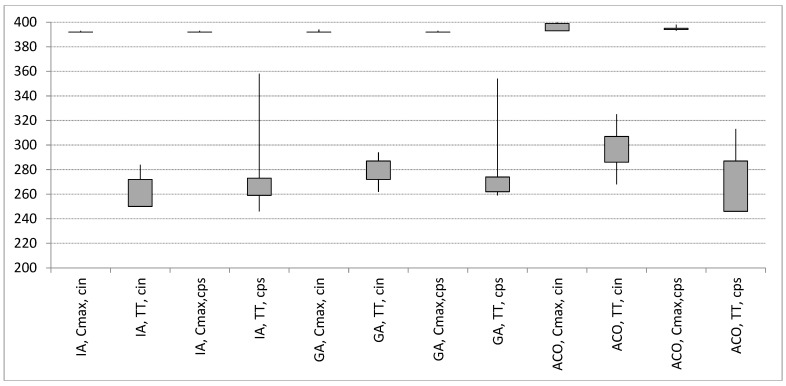
Cmax (Cmax) and TT (TT) obtained by the ACO, GA and IA for changing: number of iterations (cin), size of population (cps).

In Figure 7, 25th quartile and 75th quantile, minimum and maximum values of the objective functions: Cmax (Cmax) and TT (TT) obtained by the ACO, GA, and IA for changing: number of iterations (cin), size of population (cps) are presented.

The IA generates the best schedules with the lowest variability with equal inputs. The best schedule was achieved for: Cmax = 393, TT = 255 using the ACO, Cmax = 392, TT = 246 using the IA, and Cmax = 392, TT = 259 using the GA.

### 3.3. Modeling, Simulation and Results

**Figure 8:**
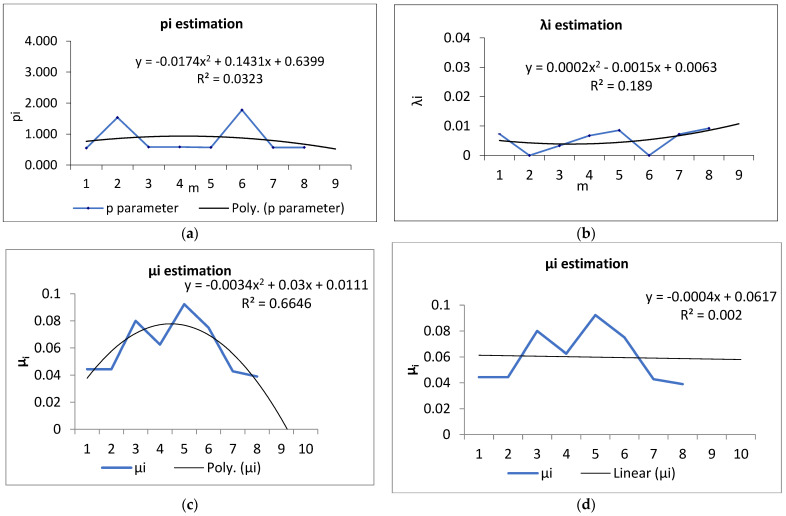
Parameters of MTTF and MTTR estimation for the FlexSim simulation: (**a**) $\hat{p_{i+1}}$; (**b**) $\hat{\lambda_{i}}$; (**c**) $\hat{\alpha_{i+1}}$ with the polynomial trend function; (**d**) $\hat{\alpha_{i+1}}$ with the linear trend function.

The input data for the FlexSim simulation program were 50 schedules received by the IA, and the MTTF of the washing machine obtained using the Maximum likelihood approach. Shape parameter $\hat{p_{i}}$ for *i*th successive month $\langle (i-1)T,iT){,}_{}i=1,\ldots ,8$ was {0.547, 0.534, 0.582, 0.582, 0.567, 1.777, 0.567, 0.567}. Scale parameter $\hat{\lambda_{i}}$ for *i*th successive month was {0.007302573, 2.87523E-07, 0.003295615, 0.006741512, 0.008557754, 1.23601E-07, 0.007191757, 0.009227353}. The predicted parameters are $\hat{p_{i+1}}=0.5184$ and $\hat{\hat{\lambda_{i}}=0.02142}$, and, finally, trend functions describing the empirical data on failure-free times are presented in Figure 8a for parameter $\hat{p_{i+1}}$ and Figure 8b for parameter $\hat{\lambda_{i}}$. The predicted MTTF is 3110 mins. 

Mean Time of Repair MTTR is described by the exponential distribution (11). Parameter $\hat{\alpha_{i}}$ of the exponential distribution for ith successive month $\left[\left(i-1\right)T,iT\right){,}_{}i=1,\ldots ,8$ is {0.044, 0.0444, 0.08, 0.0625, 0.0923, 0.075, 0.0428, 0.039}. The predicted parameter $\hat{\alpha_{i+1}}=0.0057$ was obtained for the polynomial trend function (Figure 8c) and $\hat{\alpha_{i+1}}=0.058$ was obtained for the linear trend function (Figure 8d). The predicted MTTR was 17.21 mins based on the linear trend function. 

**Figure 9:**
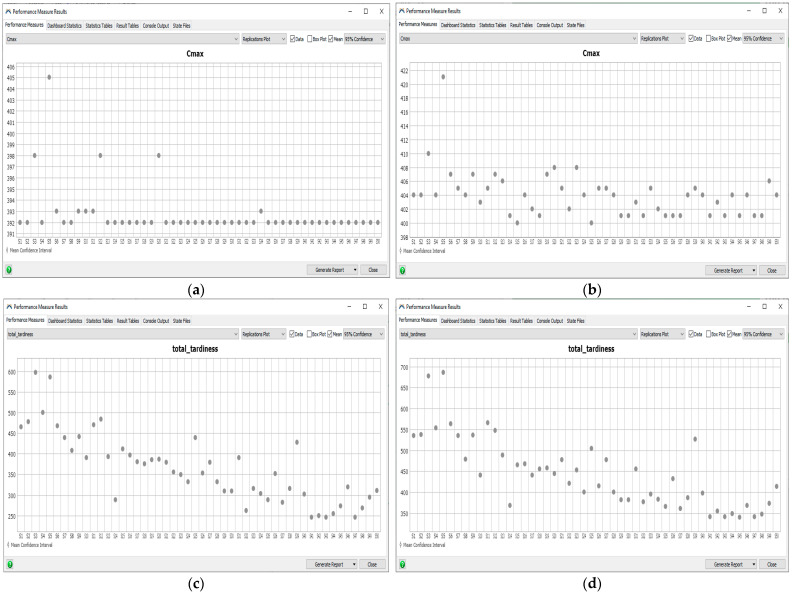
Cmax (**a**,**b**) and TT (**c**,**d**) of schedules (schedules S1–S50) without (**a**,**c**) and with predictive maintenance (**b**,**d**).

**Figure 10:**
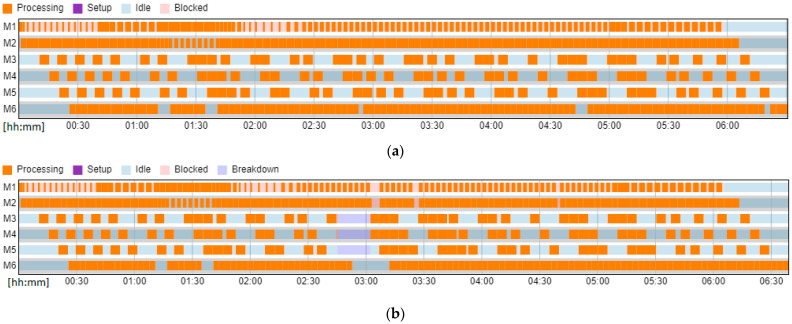
The Gantt chart of the best schedule achieved by the FlexSim (**a**) without and (**b**) with predictive maintenance.

Experiments were carried out and the schedules described by Cmax and TT criteria were presented in Figure 9. For the Cmax minimization problem, the best schedules were achieved with Cmax = 392 (Figure 9a). It should also be noted that the results determined using the Digital Twin in the form of a simulation model also take into account the subsystems of handling and the availability of operators and their travel times, which is, usually, omitted in conventional scheduling systems based on analytical models. For the TT minimization problem, the best schedule was achieved for the forty-first sequence of tasks {1 11 3 7 5 6 9 0 2 4 8 10} with TT=246 (Figure 9c). The forty-first sequence is also the best, including the results achieved by the IA. The Gantt chart of the best schedule generated is presented in Figure 10a.

The Gantt chart of the best schedule with predictive maintenance is presented in Figure 10b. Taking into account the technical inspection of the washing machine is very important for managers when negotiating the deadlines for the execution of production orders to ensure their timeliness.

## 4. Discussion

The integration of optimization and simulation is important for managers to take full advantage of limited production line resources and ensure on-time production. For each produced Digital Twin, it is necessary to study the set of parameters of each algorithm: GA, IA, and ACO. 

The selection of the *q*_0_ parameter is crucial and can improve the quality of solutions generated by the ACO. The *q*_0_ parameter determines the choice of exploration or exploitation by each ant. In the analyzed case study, the value of the parameter *q*_0_ = 0.1 means that almost all ants (90%) discover new areas. The best schedules are achieved if only 10% of all ants have a chance to use the existing schedules. 

The value of relative importance between the pheromone trace and the reciprocal of the distance *β* = 0.3 means that the pheromone trace (exponent = 1) is more important than the reciprocal of the distance (exponent = 0.3). The pheromone pairing coefficient *ρ* = 0.9 means that global ACO searching ability improves; at the same time, the speed of convergence decreases.

It has been observed that the chance of the ACO reaching a non-delayed schedule increases as the number of ants increases. The same phenomenon was noticed for the increase in the number of iterations. An increase in the number of ants and iterations results in a worsening of the Cmax. The phenomenon can be explained by the contradiction of the criteria: Cmax and TT. Considering the number of iterations, a sufficient (minimum) number is 90 for IA and GA and 160 for ACO to identify the best schedule.

The best schedule was achieved by IA (Figure 7). Taking into account the computation time, the presented IA algorithm is less time-consuming due to the structure allowing for the parallel search process to be carried out separately for each objective function. In order to speed up the search process using the ACO algorithm, it is proposed to divide the population into two separate ones for each objective function. 

Scientists outdo each other in search of effective methods of solving problems of planning production tasks, inspired by the world of nature. The presented ACO algorithm was compared with GA [37] and IA [38]. The presented optimization methods find similar solutions, but, taking into account the simulation time, the IA is better. At the operational level, the time to find a solution is relatively short, which, in turn, results in a short time to update the simulation model [19,20,21]. The main contribution is made to the integration and takes in the advantage of acquisition data, optimization, prediction, and simulation, instead of focusing solely on the optimization aspect.

For each produced Digital Twin, it is necessary to predict the reliability parameters of the rescripted resources in order to set stable deadlines for the implementation of production tasks. The integration of the prediction and simulation is also important for managers to take full advantage of limited production line resources and ensure on-time production.

The predicted parameter describing repair time was obtained for the polynomial trend function and the linear trend function (Figure 7c,d). Although the polynomial trend described the historical data with great accuracy, it yielded a forecast of MTTR = 175.43. The forecast for the next month differed significantly from historical data; therefore, it was necessary to take into account the more reliable forecast using a linear trend.

**Figure 11:**
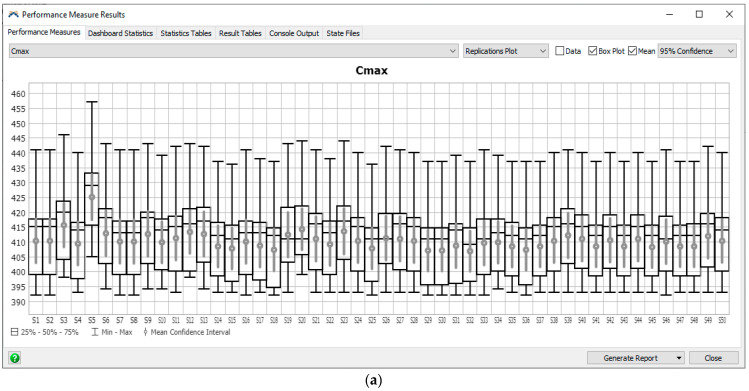

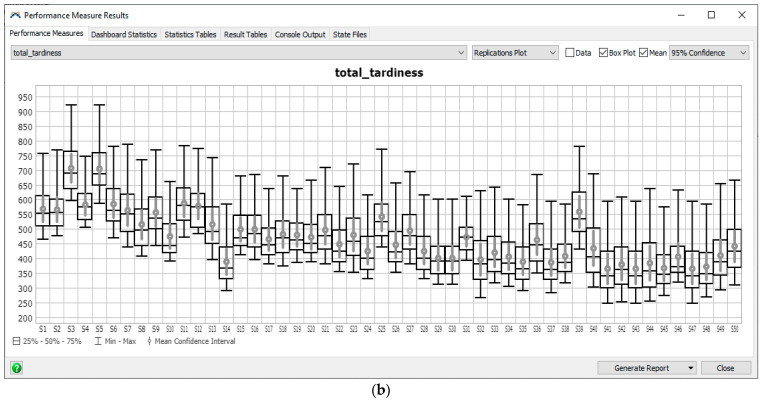
Cmax (**a**) and TT (**b**) of schedules (schedules S1–S50) obtained using the IA and verified in FlexSim.

Industry 4.0 promotes the integration of simulation tools, historical data analysis, as well as artificial intelligence and machine learning methods, which allows the company to obtain measurable benefits presented in the article. In contrast, without optimization and prediction methods, the simulation tool itself gives too much information scatter, which makes it difficult for the manager to make a decision. As an example, in each schedule, the MTTR parameter described by the exponential distribution with $\hat{\alpha_{i+1}}=0.058$ represents maintenance time (Figure 11). 

Experiments were performed for 15 simulation replications for each sequence achieved by the IA (schedules S1 through S50) with the repair time described by the exponential distribution with the mean of 17.2 and the standard deviation of 17.6. The best schedule was obtained for the forty-first sequence of tasks {1 11 3 7 5 6 9 0 2 4 8 10} with a variable duration of the maintenance. In the worst-case scenario of the best schedule, the TT was 593 min.

## 5. Conclusions

In this paper, the concept of the smart factory operation was presented along with the idea of data exchange architecture, simulation creation, performance optimization, and predictive analysis of the production process conditions. The Digital Twin for the hybrid flow shop from the automotive industry was presented as a case study. The ACO algorithm was developed for multi-criteria scheduling problems in order to obtain a production plan without delays and maximum resource utilization. The ACO was compared to the IA and GA. The best schedules were achieved with low computation time using the IA for the Digital Twin. Mean Time To Failure and Mean Time of Repair were predicted for a real case study of an electric steering gear production line in order to build predictive schedules.

The benefits of the proposed approach for Industry 4.0 are: 

By predicting the reliability parameters of the limited resources of the Digital Twin, stable deadlines for the implementation of production tasks are achieved. 

Thanks to the construction of a smart factory, online data acquisition, and failure detection, a quick reaction of the production system to a disruption is possible.

Through the integration operation, together with the creation of simulation, performance optimization and predictive analysis of the conditions of the production process, the best possible answer is proposed.

The subject of further research will be the development of data aggregation and exchange methods to support the optimization of material and information flows in all areas of production systems. Recently, algorithms where the global search process is not random, but moves according to the position of the best individuals in the population, such as the sparrow search algorithm, gray wolf optimization algorithm, and Harris hawks optimizer, have been enjoying great interest. In the future, the proposed approach will be developed taking into account the new trends in optimization methods. In addition, machine learning methods will be used to predict robustness and stability criteria for flow shop and hybrid flow shop problems.

## Figures and Tables

**Table 1:** Washing machine maintenance data collected.

No. Order	Problem	Idle Time	Failure Mode	Date	Failure Time	Maintenance Activities
6….7	locked tool #2	30	shavings in the tool feeder	01.03	6:20 a.m.	cleaning the magazine
6…7	filter filling level	20	the mat has been removed from the roll	15.03	9:15 a.m.	the mat pulled into the rollers was pulled out
6…2	Tool changer failure	20	dirty sensor with shavings	20.03	11:00 a.m.	cleaning the tool magazine
6…9	problem starting the machine	25	no air	28.03	6:30 a.m.	unlocking the air valve, starting the machine, testing
6…3	camera problem	20	changing the position of the parts relative to the positions set in the camera	19.04	7:30 a.m.	correction of the tool search area in the camera program
	…		…		…	

## Data Availability

Not applicable.
